# Genomics in Eels — Towards Aquaculture and Biology

**DOI:** 10.1007/s10126-012-9444-5

**Published:** 2012-04-17

**Authors:** Yuki Minegishi, Christiaan V. Henkel, Ron P. Dirks, Guido E. E. J. M. van den Thillart

**Affiliations:** 1 Institute of Biology Leiden, Leiden University, P.O. Box 9505, 2300 RA Leiden, The Netherlands; 2 ZF-screens B.V., Niels Bohrweg 11, 2333 CA Leiden, The Netherlands

**Keywords:** *Anguilla*, Genome, Next generation sequencing (NGS), Aquaculture

## Abstract

Freshwater eels (genus *Anguilla*), especially the species inhabiting the temperate areas such as the European, American and Japanese eels, are important aquaculture species. Although artificial reproduction has been attempted since the 1930s and large numbers of studies have been conducted, it has not yet fully succeeded. Problems in eel artificial breeding are highly diverse, for instance, lack of basic information about reproduction in nature, no appropriate food for larvae, high mortality, and high individual variation in adults in response to maturation induction. Over the last decade, genomic data have been obtained for a variety of aquatic organisms. Recent technological advances in sequencing and computation now enable the accumulation of genomic information even for non-model species. The draft genome of the European eel *Anguilla anguilla* has been recently determined using Illumina technology and transcriptomic data based on next generation sequencing have been emerging. Extensive genomic information will facilitate many aspects of the artificial reproduction of eels. Here, we review the progress in genome-wide studies of eels, including additional analysis of the European eel genome data, and discuss future directions and implications of genomic data for aquaculture.

## Background

Freshwater eels, genus *Anguilla*, consist of 19 species and subspecies, and inhabit from tropical to temperate areas all over the world, except for the coastal lines of the South Atlantic and eastern Pacific oceans (Ege 1939; Watanabe et al. 2009). All anguillid species have catadromous life histories, in which spawning occurs in the sea, and eggs and larvae (called leptocephali) are transported by warm ocean currents to coastal recruitment areas. When they reach there, they metamorphose from larva to juvenile, called glass eel, and migrate upstream. After several years in their growth habitats in freshwater rivers, estuaries and lakes, they start downstream migration and go back to their natal spawning areas in the ocean for reproduction (Tesch 2003; Tsukamoto 2009). The eel migration, which for some species spans thousands of kilometres, has been a great mystery until recently, when the spawning area of the Japanese eel (*Anguilla japonica*) was pinpointed near the Western Mariana Ridge (Tsukamoto 2006; Tsukamoto et al. 2011). This unique ecology of eels has instigated many scientific questions, and thus intensive research has been conducted to reveal their biology.

Apart from this biological particularity, eels have also received much economical attention because they are one of the most commercially important aquatic species in Asia, Europe, the United States and New Zealand. The global production in 2009 was approximately 284,000 tons, of which 97 % is derived from aquaculture (FAO Fisheries and Aquaculture Information and Statistics Service). Since there is no artificial eel seed for aquaculture, eel resource is currently fully dependent on the natural population: glass eels annually recruiting to continental areas from their spawning ground. However, glass eel catch has been drastically declining over the last four decades (Dekker et al. 2003; ICES 2008). In fact, the population of the European eel *A. anguilla* has collapsed to the point where it is now listed as a critically endangered species (IUCN 2008). Strict stock management is implemented at the European Union level, which includes not only regulation of glass eel catches but also adult eel fishing (ICES 2010). Accordingly, there is an urgent need for success in artificial reproduction to compensate for the shortage of the eel stock.

Artificial reproduction of eels has been attempted since the 1930s. Based on the first experiments in the European eel (Fontaine 1936; Fontaine et al. 1964), artificial sexual maturation of eels is induced in *A. anguilla*, *A. rostrata*, *A. japonica*, *A. dieffenbachii* and *A. australis* by repetitive injections of gonadotropic hormone (human chorionic gonadotropin) and pituitary homogenate (Ohta et al. 1997; Lokman and Young 2000; Lokman et al. 2001; Palstra and van den Thillart 2009; Oliveira and Hable 2010). Nonetheless, for a large-scale production for glass eels, the current technique is not feasible. Even in the Japanese eel, for which artificial reproduction is the most successful among the congeners, maturation, gametogenesis and spawning have never occurred spontaneously, and the mortality is still high (Kagawa et al. 2005). In other species, eggs and larvae have been artificially produced, but have not reached further development, as no suitable food for larvae has been discovered and rearing conditions including temperature, salinity and light regime have to be optimized (Palstra et al. 2005; Oliveira and Hable 2010). Considering a rapid population decline, other approaches are definitely needed to facilitate eel reproduction.

As a result of recent remarkable advances in computational and sequencing technology, i.e., Next generation sequencing (NGS), genomic-scale data have become more easily available and accessible for many aquatic organisms, even non-model species. For instance, numerous numbers of studies have been carried out based on the draft genome of zebrafish *Danio rerio* to investigate the mechanisms of various biological features including early embryogenesis, spermatogenesis, oogenesis, and even human disease (e.g., Zeng and Gong 2002; Newman et al. 2010; Aanes et al. 2011). In eels, the draft genome of the European eel has been recently determined using Illumina technology (Henkel et al. 2012) and transcriptomic data based on NGS has been emerging (Coppe et al. 2010). In addition to its value in studies of fundamental biology, genomics can provide a wealth of information on, for instance, maturation, development, responses to rearing conditions, and so on, making it a powerful tool for aquaculture.

Here, we review the current status of eel genomics, mainly focusing on the aspects relevant to aquaculture research. We first examine the traditional approaches to scan genomes such as Amplified Fragment Length Polymorphisms (AFLP) and physical linkage maps. We then review the draft genome of the European eel that has been recently reported by Henkel et al. (2012) with some additional analyses, which will be the basis of future eel genomic studies and aquaculture, and consider genome-wide gene expression profiling as a possible application of the eel genome sequence. Finally, we discuss the implications and possible future directions of eel genomics.

## Genome Scanning

Traditionally, various regulatory mechanisms have been investigated using classical molecular biological methods such as molecular cloning, recombinant protein purification and laboratory observations, and the amount of genetic variation between and within taxa has been examined using molecular markers like mitochondrial DNA and microsatellites. In recent years, genome scanning approaches such as AFLP and single nucleotide polymorphisms (SNP) discovery have been used to obtain genetic variations at a genomic level. In eels, one of the most common genome scanning approaches is probably AFLP. Ishikawa et al. (2004) and Gagnaire et al. (2011) used this technique to survey the genetic structure of the giant mottled eel *A. marmorata*, and described the footprint of natural selection and the detailed genetic connectivity between populations. These genome scan studies clearly show their much higher resolution powers to detect subtle genetic variations and signals of evolution compared to classical molecular markers.

Albert et al. (2006) used the same method to determine the status of hybrids of the European and American eels, which occur naturally only in Iceland (Avise et al. 1990). This study revealed the existence of further hybrid generations of F1 there, suggesting that hybrids can reproduce successfully (Albert et al. 2006). This finding serves a warning to eel aquaculture, because live stocks have been traded, for example the glass eels of the European species were imported into Japan, and the presence of the introduced anguillid species in Japanese natural waters has been reported (Tabeta et al. 1977; Zhang et al. 1999; Aoyama et al. 2000; Okamura et al. 2002). Furthermore, it is possible to induce interspecific hybridization in other species than the European and American eels (Okamura et al. 2004; Burgerhout et al. 2011), even though it is not known whether those artificial hybrids are fertile. Genome scanning methods are therefore potentially more appropriate to evaluate genetic contamination, i.e., genetic “health” of aquaculture stocks and natural populations in eels.

Recently, Nomura et al. (2011) constructed a physical linkage map of the Japanese eel using AFLP and microsatellite markers. Genetic linkage maps are quite useful for aquaculture since they enable the analysis of quantitative traits loci (QTL) that determine commercially important traits such as spawning time and behaviour (rainbow trout, Leder et al. 2006; Colihueque et al. 2010), body growth and sex determination (rainbow trout, Wringe et al. 2010; Arctic charr, Küttner et al. 2011; gilthead sea bream, Loukovitis et al. 2011), hatch timing (coho salmon, McClelland and Naish 2010), embryonic development rate (rainbow trout, Robison et al. 2001), thermal tolerance (rainbow trout, Perry et al. 2005), disease resistance (rainbow trout, Baerwald et al. 2011; Atlantic salmon, Houston et al. 2008), and stress response (sea bass, Massault et al. 2010). Compared with those species, QTL mapping of eel species has not made progress so far due to the unavailability of F2 and backcrossed animals. The genetic linkage map by Nomura et al. (2011) will be the basis of future QTL studies of eels to discover the crucial traits for eel aquaculture like developmental and growth rate, timing of metamorphosis, quality of eggs and sperm, fertilization rate, mortality, immune sensitivity and tolerance to virus infections and diseases, quality of fish meat, and so on.

Other new methods using NGS such as reduced-representation sequencing and restriction site associated DNA sequencing (RAD-seq) have been also developed, and effectively applied in genome-wide SNP identification and linkage map construction (see Andolfatto et al. 2011; Davey et al. 2011; Rowe et al. 2011 for review). In fish, RAD-seq from one single Illumina lane successfully discovered about 3,000 SNPs in rainbow and westslope cutthroat trout, showing the feasibility of this method for non-model species in a cost-efficient manner (Hohenlohe et al. 2010). Although genome scan studies in eels are at this moment still few in number, these techniques will facilitate the production of useful information for QTL mapping and phylogeography, and also various biomarkers such as broodstock selection.

## Draft Genome Sequence

In fish, as in other taxa, full genomic sequencing was first performed in model species such as zebrafish, medaka, and fugu (Aparicio et al. 2002; Kasahara et al. 2007; Kai et al. 2011). Moreover, 63 genome projects are currently ongoing in fish such as coelacanth and Atlantic salmon (see also Davidson et al. 2010) according to the database (GOLD; http://genomesonline.org, as of February 20, 2012). Those genomes have been widely used as a reference for studies of other fishes, e.g., bacterial artificial chromosome (BAC) end sequence analysis of rainbow trout, gilthead sea bream and common carp, which are also commercial species (Genet et al. 2011; Kuhl et al. 2011; Xu et al. 2011). Although the published genomes of those species have already contributed to major progress in fish biology, the genome information is relatively limited in relation to the highest species diversity of fishes among vertebrates and their economical importance.

In 2012, Henkel et al. (2012) employed Illumina sequencing technology and determined the draft genome of the European eel (www.eelgenome.org). This is the first report of the entire genome sequence of the genus *Anguilla* and the order Anguilliformes. The assembled genome consists of contigs with a typical scaffolds length (N50) of 1.7 Kbp. The majority of contigs was further arranged into 186,000 genomic scaffolds with an N50 of 77.6 Kbp, yielding a final assembly of 923 Mbp. It turns out that 179 Mbp of contigs were not included in larger scaffolds because of their small sizes or highly repetitive sequences. These numbers agree well with the genome size (1.1 Gbp) estimated based on flow cytometry (Henkel et al. 2012), suggesting the assembly of the European eel genome is reasonably complete.

Subsequently Henkel et al. (2012) investigated the *Hox* gene complement in the genome of the European eel and found that the eel genome contains the almost complete duplicate set of *Hox* genes established at the teleost specific genome duplication (approximately 300 million years ago), whereas many of these *Hox* genes have been lost in the higher fish groups during evolution. This finding was consistent with a previous study that described *Hox* genes of the Japanese eel (Guo et al. 2010), and thus the authors concluded that the eel genome retains primitive traits in comparison with other teleosts. Because of this novel genome organization and the rather basal phylogenetic position of eels among teleosts (Miya et al. 2003; Inoue et al. 2004), this study suggests that the eel genome can be a useful source for comparative genomics studies.

**Fig. 1:**
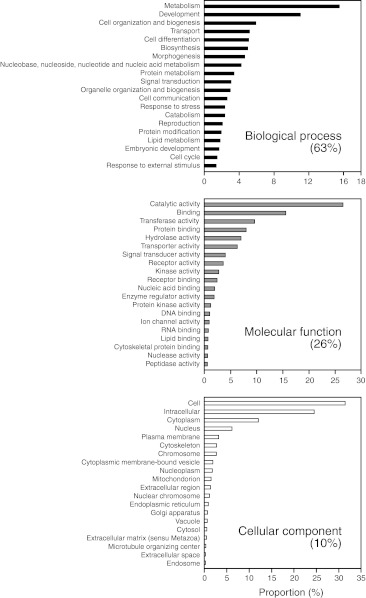
Gene ontology category distribution in each of the three main GO domains (biological process [*BP*], molecular function [*MF*] and cellular component [*CC*]) of the European eel. 10,242 out of a total of 186,116 GO terms that were significantly provisionally annotated by Henkel et al. (2012) were first assigned to the three ancestral categories (BP, MF, CC), and subsequent analysis was performed in each category. The percentage indicated just below the three domain names presents the proportions of terms assigned to a category relative to the total GO terms. Top 20 fractions are shown in percentage in the graph for each category

The study by Henkel et al. (2012) resulted in about 46,000 predicted genes in the European eel genome. In this work, we show the result of the gene ontology (GO) analysis using 16,402 genes (35.7 %) of the European eel, which were provisionally annotated by Blast2GO (Conesa et al. 2005). Using a web-based GO analysis tool CateGOrizer (Hu et al. 2008), all 186,116 GO terms found in those 16,402 genes were first assigned to the main three GO domains (biological process [GO:0008150], molecular function [GO:0003674] and cellular component [GO:0005575]), and then further GO category distributions were investigated in each of the three categories based on GO Slim2 terms, which is the simplified version of the whole GO terms (Hu et al. 2008). The first assignment to the three root categories found more than 60 % of the terms in biological process, 25.8 % in molecular function, and 10.1 % in cellular component (Fig. 1). A total of 111 GO terms (1.1 %) were not assigned to any of the three categories, which could be due to unsynchronized GO databases. Subsequent analysis in each of the main three domains showed high proportion of metabolism [GO:0008152] and development [GO:0007275] in biological process, catalytic activity [GO:0003824] and binding [GO:0005488] in molecular function, and cell [GO:0005623] and intracellular [GO:0005622] in cellular component, and again found some unassigned GO terms (12.8 % in biological process, 1.0 % in molecular function and 4.0 % in cellular component) (Fig. 1). Since the limited availability of reliable in silico annotation in the first draft genome of the European eel prevents the comparison of GO term distribution with other fish species, the characterization of the eel genome and its functional analyses will be the subjects of future studies.

Additionally another genome sequencing project of eels is in progress at this moment, the Japanese eel (Henkel et al. submitted). This will make a big step in eel genomics, because it will allow comparative genomics studies of eels, which will provide various kinds of useful information in aquaculture and other biological fields. For instance, genetic diversity is one of the serious issues in aquaculture and conservation, since it tends to decrease in populations whose effective population sizes are small like the ones in aquaculture. Loss of genetic diversity has been reported in commercial hatcheries and endangered natural broodstocks of barramundi, Arctic charr, brook charr and Atlantic salmon (Primmer et al. 1999; Frost et al. 2006; Lage and Kornfield 2006; Marie et al. 2010), and only few studies have examined the temporal change in genetic diversity in eels (Han et al. 2008, 2010; Pujolar et al. 2011). However, their assessments of genetic diversity were based on several microsatellite loci, which are not necessarily representing the actual genetic diversity of a population, while genome information should give a whole picture of genetic diversity.

## Gene Expression Profiling

Phenotypic changes are genetically regulated, specifically, by switching on/off genes associated with sets of QTL as mentioned above. Accordingly, to capture the background mechanisms of biological phenomena and responses to various stimuli, gene expression needs to be investigated on a genome-wide scale. For this purpose, expressed sequence tags (EST) have been employed in many fish species like Atlantic salmon, gilthead sea bream, European seabass and Senegalese sole (Cerdà et al. 2008; Koop et al. 2008; Cepeda et al. 2011; Louro et al. 2011), which provides rich genetic sources for future genomic scale studies like microarrays. In contrast, EST has not been a very common tool in eels and the study by Miyahara et al. (2000) is the only one to our best knowledge. They generated 196 EST from a cDNA library of spleen of the Japanese eel, 46 % of which showed no homology to known genes, and found that six identified EST among the rest (54 %) were similar to those of the Japanese flounder in their previous study, suggesting that the constructed EST can be useful for comparative gene expression studies in fish spleen (Miyahara et al. 2000).

Another popular gene expression profiling approach is cDNA microarrays. Since the catadromous life history of eels requires adaptations to different water environments, i.e., seawater and freshwater, the mechanisms of osmoregulation have been investigated from various aspects such as specific hormones and their receptors (Kozaka et al. 2003; Wilson et al. 2004; Yuge et al. 2006), membrane lipids (Hansen and Grosell 2004) and morphological changes of chloride cells (Seo et al. 2009). Kalujnaia et al. (2007a, b) constructed a microarray comprising 6,144 cDNAs from brain, gill, intestine, and kidney libraries and identified 229 differentially expressed clones between groups that were acclimatized in freshwater and seawater. Subsequent sequencing analysis attributed 95 out of 229 to known genes related to detoxification, energy metabolism and respiration, cell protection and immune system, signal transduction, etc. Major findings of these microarray studies were the identification of a number of genes that were already known to be involved in osmoregulation, but also the discovery of many other genes that were not previously found to be associated with ion or water transport (Kalujnaia et al. 2007b).

With recent rapid advances in sequencing technologies, i.e., the NGS, complete sets of transcripts in a population of cells/tissues (transcriptome) can be accurately determined and quantified. This method is termed RNA-seq. In eels, the first RNA-seq was reported for wild-caught glass eels (Coppe et al. 2010). They analyzed a normalized cDNA library obtained from a pooled sample of 18 glass eel heads using 454 FLX Titanium sequencing, and ended up with more than 300,000 reads that were assembled in nearly 20,000 contigs, about 36 % of which were similar to known protein/nucleotide sequences (Coppe et al. 2010). In the gene ontology analysis of this transcriptome, more than 50 % of these could be mapped to a GO term of biological process. The sequencing technology and strategy employed in their study precluded reliable quantification of the obtained transcript contigs, and the lack of comparative samples has limited biological interpretations of the result in their study.

However, the data can provide a rich resource for the discovery of new genes and the identification of biomolecular markers. For instance, it has been well described that sexual maturation of eels is associated with a variety of changes such as internal hormone levels (Dufour et al. 2001 for review; Jeng et al. 2007), body coloration (Sinha and Jones 1975; Pankhurst 1982; Haro 2003; Okamura et al. 2007), enlargement of pectoral fins and eye diameter (Durif et al. 2005; van Ginneken et al. 2007), degeneration of the digestive tract (Pankhurst 1982), and morphology and number of chloride cells in the gill (Seo et al. 2009). If differentially expressed genes corresponding to those phenotypic changes are identified by transcriptome analyses, they can be molecular probes for maturation stages and will help understand the whole genetic mechanism of maturation in eels. Likewise, the same methodology will be applicable for other biological features of eels.

## Conclusions: Implications and Future Directions

Studies employing genomic resources have become increasingly popular in recent years. In eels, genomics is still at a very early stage where the basic data are being accumulated, but have not yet been applied for other functional studies like proteomics. However, the draft genome sequence of the European eel is now available, and that of the Japanese eel will be disclosed very soon (Henkel et al. submitted), which provides a solid basis for comparative genomics in eels. Those genome data can be also useful to improve transcriptome data analysis, as Henkel et al. (2012) showed by identifying the *Hox* genes using the embryonic transcriptome of *A. australis*. Moreover, the costs of quantitative RNA-seq have come down rapidly and thus it is now replacing the less versatile cDNA microarrays. Consequently, it is becoming feasible to study the gene expression patterns and regulatory mechanisms of specific phenomena like maturation and development. In summary, eel genomics is quite rapidly growing up. We now have more choices and approaches to observe genetic backgrounds and mechanisms of various phenotypic traits than ever before. However, this does not necessarily mean that those high-throughput techniques based on NGS are always the better choice; this fully depends on the research objectives. As the field of eel genomics matures, it will improve aquaculture techniques by modifying protocols based on information obtained from transcriptome analyses and functional genomics, and open up perspectives on fundamental biological questions in eel research.
